# Supplementary material for: The flow responsive transcription factor Klf2 is required for myocardial wall integrity by modulating Fgf signaling
Source: eLife. 2018 Dec 28;7:e38889. doi: 10.7554/eLife.38889 (PMC6329608; doi:10.7554/eLife.38889)
Supplement: Figure 6—source data 1. [file elife-38889-fig6-data1.docx]

**Figure 6-source data 1**

| Transcript ID | Gene | *klf2* WT | *klf2* Mut | *klf2* Mut/WT ratio |
| --- | --- | --- | --- | --- |
| ENSDART00000075519 | *aldh1a2* | 4408 | 6998 | 1,58 |
| ENSDART00000141212 | *cyp26a1* | 20 | 25 | 1,23 |
| ENSDART00000041728 | *cyp26a1* | 161 | 172 | 1,07 |
| ENSDART00000077809 | *cyp26c1* | 37 | 29 | 0,79 |
| ENSDART00000110347 | *cyp26b1* | 4906 | 1635 | 0,33 |
