# Supplementary material for: The flow responsive transcription factor Klf2 is required for myocardial wall integrity by modulating Fgf signaling
Source: eLife. 2018 Dec 28;7:e38889. doi: 10.7554/eLife.38889 (PMC6329608; doi:10.7554/eLife.38889)
Supplement: Figure 6—source data 2. [file elife-38889-fig6-data2.docx]

| Transcript ID | Gene | *klf2* *WT* | *klf2* *Mut* | *klf2 Mut/WT* ratio |
| --- | --- | --- | --- | --- |
| ENSDART00000137875 | *dhh* | 125 | 103 | 0,82 |
| ENSDART00000122719 | *ihhb* | 49 | 40 | 0,81 |
| ENSDART00000099204 | *shha* | 78 | 60 | 0,76 |
| ENSDART00000149395 | *shha* | 157 | 118 | 0,75 |
| ENSDART00000081660 | *ihha* | 638 | 457 | 0,71 |
| ENSDART00000085051 | *hhip* | 284 | 114 | 0,40 |
| ENSDART00000053871 | *dhh* | 79 | 5 | 0,06 |
| ENSDART00000049151 | *gli1* | 20 | 3 | 0,18 |

**Figure 6-source data 2**
