# Supplementary material for: The flow responsive transcription factor Klf2 is required for myocardial wall integrity by modulating Fgf signaling
Source: eLife. 2018 Dec 28;7:e38889. doi: 10.7554/eLife.38889 (PMC6329608; doi:10.7554/eLife.38889)
Supplement: Figure 6—source data 3. [file elife-38889-fig6-data3.docx]

**Figure 6-source data 3**

| Transcript ID | Gene | *klf2* WT | *klf2* Mut | *klf2* Mut/WT ratio |
| --- | --- | --- | --- | --- |
| ENSDART00000013534 | *fgfr3* | 1249 | 1070 | 0,85 |
| ENSDART00000125883 | *fgfr3* | 1285 | 1101 | 0,85 |
| ENSDART00000098269 | *fgfr2* | 1532 | 1159 | 0,75 |
| ENSDART00000080916 | *fgfr2* | 1627 | 1211 | 0,74 |
| ENSDART00000080914 | *fgfr2* | 1679 | 1214 | 0,72 |
| ENSDART00000100286 | *fgfr4* | 2731 | 1923 | 0,70 |
| ENSDART00000127119 | *fgfr1a* | 310 | 208 | 0,66 |
| ENSDART00000147742 | *fgfr1a* | 780 | 477 | 0,61 |
| ENSDART00000074774 | *fgfr1a* | 410 | 233 | 0,56 |
| ENSDART00000110754 | *Fgf3* | 1995 | 910 | 0,45 |
| ENSDART00000098283 | *fgf4* | 87 | 48 | 0,55 |
| ENSDART00000060051 | *Fgf14* | 141 | 71 | 0,50 |
| ENSDART00000029630 | *fgf17* | 413 | 251 | 0,60 |
| ENSDART00000040013 | *myl7* | 77279 | 70540 | 0,91 |
| ENSDART00000147074 | *fli1a* | 455 | 432 | 0,94 |
| ENSDART00000098696 | *nrg2a* | 567 | 587 | 1,03 |
| ENSDART00000087339 | *cdon* | 4788 | 3159 | 0,65 |
| ENSDART00000054460 | *egr1* | 10856 | 5705 | 0,52 |
